# A comparison of the physical demands generated by playing different opponents in basketball friendly matches

**DOI:** 10.5114/biolsport.2024.129474

**Published:** 2023-08-08

**Authors:** Takeshi Koyama, Jun Nishikawa, Kaishi Yaguchi, Takayuki Irino, Akira Rikukawa

**Affiliations:** 1 Department of Physical Education, Tokai university, Japan; 2 Graduate School of Physical Education, Tokai university, Japan; 3 Department of Sports Promotion Center, Tokai university, Japan

**Keywords:** Team sports, Exertion, Match analysis, Load monitoring, Game demands

## Abstract

This study aimed to compare the physical demands of playing opponents of different skill levels in basketball. Eighteen men’s college basketball players wore accelerometers to measure the relative accumulated acceleration load (AAL), estimated equivalent distance, and frequencies of sprint, jump, and exertion events during games against professional teams (Pro), teams at the same competition level (Collegiate), and teams comprising intra-team members in practice games (Scrimmage). Internal responses were calculated using the relative rating of perceived exertion (sRPE). A repeated measures analysis of variance, Bonferroni post-hoc tests, and standardized Cohen’s effect sizes were calculated to compare the physical demands and internal responses across matches played against different levels of opponents. The results showed that in the game against the Pro, AAL (arbitrary units), sprint events (cases per min), and exertion events (cases per min) were significantly (*p* < .05) higher than those in games against the Collegiate and Scrimmage teams. As the competitive level of the opponents increased, the relative external load of the participants also increased. Conversely, internal responses measured using sRPE were lower after games against the Pro than those against the Collegiate. Internal and external loads may vary from each other depending on contextual factors.

## INTRODUCTION

Training load is defined as the amount of stress on an individual from a single or multiple training sessions over time [1]. Understanding the influence of training load outcomes on sports performance and injury prevention is vital in sports medicine; it is also important for strength and conditioning coaches and sports scientists [1]. Therefore, implementing techniques to monitor athletes’ responses to training stimuli is pertinent to team sports coaches and conditioning professionals [2]. Training load can be organized as “external load,” the work completed by an athlete [3], and internal load, the psycho-physiological responses of the athlete to the exercise demand [4]. The term “external load” refers to any external stimulus applied to athletes that is measured independently of their internal characteristics [5]. The external load will result in physiological and psychological responses in each “individual characteristic,” following interaction with, and variation in several other biological and environmental factors [6]. This individual response is referred to as the “internal load” [1]. In other words, the training outcome is the consequence of the internal load determined by individual characteristics, such as genetic factors and previous training experience, and the quality, quantity, and organization of the external load [6]. In recent years, accelerometers have gained interest as a practical approach for measuring the external load in team sports [1].

Basketball is an intermittent sport that, due to court dimensions, number of players, and rules (for example, ball possession time [24 s]), requires the players to perform repeated high-intensity movements, such as rapid changes in direction and cutting actions, changes in speed over short distances, contacts (e.g., post-ups, screens, and box outs), or run-to-jump actions occurring between different locomotor demands (e.g., standing, walking, running, and sprinting) [7]. High-intensity movements measured using accelerometers during a basketball game have been found to involve eccentric and quasi-isometric contractions, such as deceleration, landing, and physical contact [8]. Deceleration movement volumes and intensities define the extent of tissue damage [9] and subsequent injury risk [10]. Therefore, accelerometers can quantify multifaceted and complex movements and are a useful approach for monitoring the external load of basketball players [2, 11, 12]. Furthermore, differences in training load by playing position [13], game time [14], and sex [15] are also evident. Thus, understanding the physical demands of game play is a prerequisite for optimal training [3, 8].

In the clinical field of team sports such as basketball, perceived exertion is the main factor limiting human performance [16] and the most used training load monitoring tool in sports [17]. A typical example is the session rating of perceived exertion (sRPE) method, which is calculated by multiplying the rating of perceived exertion (RPE) by the duration of exercise [18]. Validity and reliability of the sRPE method in several sports and physical activities with men and women of different age categories (children, adolescents, and adults) among various expertise levels have been confirmed [17]. This method could be used as a “stand-alone” method for training load monitoring purposes [17].

An integrated approach to training load is also important, and internal and external training loads should be used in combination to provide greater insight into training stress [19, 20]. For example, athletes repeating the same session on different days may maintain the same power output for the same duration (i.e., same external load), but experience quite different internal loads (heart rate, blood lactate, and RPE) depending on their state of fatigue, emotional disturbances, recent training history, and illness [19]. A previous study on soccer has shown that match-related contextual variables, such as game outcome, game location, and score line, influence external and internal workload [20]. Game load varies by competition level in basketball [21, 22], although it is not clear how physical demands differ when the same individual plays against opponents of different competition levels. When playing against an opponent with superior performance, it is expected that there will be an external load that is not experienced in practice games within the own team or in a game against a team of the same performance level. However, no studies have thus far examined and compared these physical demands by changing the opponent for the same individual.

This study aimed to compare the physical demands of playing different opponents in basketball. Specifically, we hypothesized that the higher the competition level of opponents is, the higher are the physical demands and internal load.

## MATERIALS AND METHODS

### Participants

Eighteen men’s college basketball players (age, 19.7 ± 1.1 years [range: 18–21 years]; stature, 186.4 ± 7.6 cm; body mass, 83.9 ± 10.7 kg) were recruited for this study. The participants belonged to a division 1 top-level college league in Japan, and six of them were members of the U19 or U22 Japanese national teams. The exclusion criteria were based on a screening evaluation by a physical therapist: if a participant had a history of serious musculoskeletal injury, any musculoskeletal injury within the past 3 months, or any disorder that interfered with sensory input, musculoskeletal function, or motor function, they were excluded from participating in this study. After receiving a detailed explanation of the study benefits and risks, each participant provided written informed consent for participation. This study was approved by our university (approval number 21013) and conducted in accordance with the Declaration of Helsinki.

### Procedures

Prior to each game, inertial measurement unit (IMU) sensors (Kinexon Mobile Tag, KINEXON Precision Technologies, Munich, Germany) were positioned in a holster stitched into the shorts of each participant’s team uniform near the right posterior superior iliac spine. The holsters were constructed in collaboration with the sensor manufacturers and team equipment managers to ensure that unnecessary movement was negligible, and positioning was consistent throughout the games [23]. The closer the accelerometer is to the centre of mass, the greater is the accuracy in quantifying the physical work [24]. This placement is less susceptible to noise in the vertical vector motion resulting from upper body movements such as shoulder blade sway, arm swing, trunk flexion, and the vector magnitudes representing overall dynamic body acceleration [24]. A previous study examining the validity of this system reported the average total typical error of estimates to be 2.5% (± 1.5%) when five adult male team sport amateur athletes performed a variety of movements comprising walking, jogging, and sprinting for different distances, as well as changes of direction and jumping [25].

In this study, opposing teams were divided into three groups according to their competition level: the “Pro” were from the domestic professional league (B-league), which has a higher competition level than that of the league from which the study participants were drawn; the “Collegiate” were university teams of the same conference as the study participants’ team; and the “Scrimmage” consisted of players from the same team who play friendly matches as the study participants. Games against professionals and college students were friendly matches. Fifteen games (Pro: 5, Collegiate: 5, and Scrimmage: 5) were measured in this study, with a total of 174 data (Pro: 63, Collegiate: 57, and Scrimmage: 54). Match results were 0-5 against professionals, 3-2 against colleges, and 4-1 in Scrimmages. Data from competitions were only included if players participated in 10 minutes of live playing time [26]. Sessions were recorded throughout each game-day and were initiated and ceased at the same time for each athlete. Individual phase recordings were time stamped and segmented into warm-up, 1^st^ quarter, 2^nd^ quarter, 3^rd^ quarter, and 4^th^ quarter phases. Recording of each quarter began when the game clock started counting down and ended when the game clock reached zero. However, in this study, the analysed dataset included only the external load data obtained during the active competition minutes (i.e., during each quarter).

### Outcome measures

Across all games, microsensor data were recorded at 100 Hz via IMU devices and downloaded after each game to a personal computer for analysis using proprietary software. All system installations and calibrations were performed by the same technician before the start of the season. All matches included in this study were held at the home court of the participants. External measures included relative (min^−1^) accumulated acceleration load (AAL; arbitrary units), estimated equivalent distance (EED; m), and frequency of sprint, jump, and exertion events. AAL is a proprietary measure calculated as the accumulated rate of change in acceleration across three vectors (x, y, and z) based on the following formula:

$$\begin{array}{c} AAL=\surd [(Ac1_{n}-Ac1_{n-1}{)}^{2}+(Ac2_{n}-Ac2_{n-1}{)}^{2}\\ +(Ac3_{n}-Ac3_{n-1}{)}^{2}/100] \end{array}$$

where Ac1, Ac2, and Ac3 are the orthogonal components measured from the triaxial accelerometer and 0.01 is the scaling factor [11, 12, 20, 27]. According to previous studies, AAL has been observed to have moderate to high test-retest reliability (ICC, .94–.97; CV 3.6–9.4%) [24] and this metric has been widely used in basketball [12, 28]. EED is the sum of the estimated distances an athlete runs on a horizontal plane. The distances are derived from the velocity samples predicted from the acceleration load data recorded by the IMU [27]. The raw frequency of 100 Hz measured by the IMU was used to smooth the data to 10 Hz using a Kalman filter to identify each event. “Sprints” were identified using a threshold of 18.72 km/h and a minimum duration of 1.0 s as dictated by the proprietary software. “Jumps” were identified at a minimum dwell time of 0.4 s. “Exertions” were also identified if they maintained a minimum 4.5 G for 1.0 s as dictated by the proprietary software.

Internal measures were evaluated using the relative (min^−1^) sRPE values. sRPE was used as a perceptual indicator of the internal load based on the following formula [18]:

sRPE=RPE×Duration

where RPE = Borg’s category-ratio scale (1–10) and Duration = time in minutes.

### Statistical analysis

A priori power analysis (*α* = .05 [two-tailed], *β* = .80, *f* = .25) indicated that a minimum of 159 samples was necessary (G*Power, Version 3.1.9.6, University of Duffle Dorf, Duffle Dorf, Germany). This minimum was met in the current analysis, with 162 samples included in the analysis (Pro = 56, Collegiate = 53, Scrimmage = 53). A repeated measures analysis of variance (ANOVA) was used to assess the external and internal loads across games played against different opponents (Pro, Collegiate, and Scrimmage). Bonferroni post-hoc tests were used to determine the source of significant differences, where applicable. The effect sizes for all pairwise comparisons were determined using Cohen’s *d* with 95% confidence intervals. Cohen’s *d* was interpreted as: trivial = 0–0.19, small = 0.2–0.59, moderate = 0.6–1.19, large = 1.2–1.99, very large = 2.0–3.99, and nearly perfect ≥ 4.0 [29]. The significance level for all tests was set at *p* < .05. All statistical analyses were conducted using IBM SPSS Statistics version 26 (IBM Corp., Armonk, NY, USA) software.

## RESULTS

The number of samples included in the analysis was 164 (Pro: 56, Collegiate: 53, and Scrimmage: 53). The number of games monitored per player was 9.11 ± 3.98 (Pro: 3.22 ± 1.90, Collegiate: 2.89 ± 1.82, and Scrimmage: 3.00 ± 1.15). The external and internal outcome measures for performance against the Pro, Collegiate, and Scrimmage are presented in Table 1. The effect sizes (*d*) for all pairwise comparisons between conditions are listed in Table 2. A repeated measures ANOVA revealed significant differences between the groups for AAL, *F*_(2, 142)_ = 65.01, *p* < .001; EED, *F*_(2, 142)_ = 43.63, *p* < .001; sprints, *F*_(2, 142)_ = 31.57, *p* < .001; and exertions, *F*_(2, 120)_ = 39.42, *p* < .001). Post-hoc testing showed that playing against the Pro produced significantly higher AALs than playing against the Collegiate and Scrimmage (d = -0.50, small, *p* = .002; d = -1.74, large, *p* < .001). EED was significantly higher in games against the Pro (d = -1.31, large, *p* < .001) and Collegiate (d = -0.88, moderate, *p* < .001) compared with that in games against the Scrimmage. The number of sprint events was significantly higher in games against the Pro than in games against the Collegiate (d = -0.60, small, *p* < .001) and Scrimmage (d = -1.18, moderate, *p* < .001). Significantly more exertion events occurred during games against the Pro compared with those during games against the Collegiate (d = -0.72, moderate, *p* = .021) and Scrimmage (d = -1.85, large, *p* < .001).

**TABLE 1:** External and internal measures (mean ± SD) during a game against a professional team (vs. Pro), same competition level team (vs. Collegiate), and during an intra-team practice game (Scrimmage) in men’s college basketball players (N = 18)

Outcome Measure	Condition		

	vs. Pro (B-league)	vs. Collegiate	Scrimmage
Observations (N)	56	53	53
Duration (minutes)	28.2 ± 11.1	30.5 ± 11.8	17.3 ± 4.7*^

**External measures**			
AAL (AU per min)	12.5 ± 1.3	11.8 ± 1.5*	10.4 ± 1.1*^
EED (m per min)	94.7 ± 9.2	91.2 ± 9.9	83.1 ± 8.5*^
Sprints (cases per min)	1.64 ± 0.36	1.44 ± 0.31*	1.27 ± 0.26*^
Jumps (cases per min)	0.68 ± 0.28	0.67 ± 0.26	0.53 ± 0.20
Exertions (cases per min)	4.93 ± 0.60	4.51 ± 0.57*	3.88 ± 0.53*^

**Internal measures**			
sRPE (AU)	142.7 ± 71.8	177.7 ± 81.7*	98.6 ± 44.5*^
RPE (AU)	4.98 ± 1.29	5.72 ± 1.07*	5.50 ± 1.53^

AAL = accumulated acceleration load; EED = estimated equivalent distance; sRPE = session rating of perceived exertion; RPE = rating of perceived exertion; AU = arbitrary units;

* Significantly (*p* < .05) different from Professional level.

^ Significantly (*p* < .05) different from College level.

**TABLE 2:** Effect size (Cohen’s d with 95% confidence intervals) for pairwise comparisons between matches against the Pro, Collegiate, and Scrimmages for external and internal measures in men’s college basketball players (N = 18).

Outcome Measure	Pro vs. Collegiate				Pro vs. Scrimmage				Collegiate vs. Scrimmage			

	Cohen’s *d*		descriptor	*p* value	Cohen’s *d*		descriptor	*p* value	Cohen’s *d*		descriptor	*p* value
Duration (minutes)	0.20	(-0.18, 0.58)	*small*	0.311	-1.28	(-1.69, -0.87)	*large*	< 0.001†	-1.47	(-1.90, -1.04)	*large*	< 0.001†

**External measures**												
AAL (AU per min)	-0.50	(-0.88, -0.12)	*small*	0.002†	-1.74	(-2.19, -1.31)	*large*	< 0.001†	-1.06	(-1.47, -0.66)	*moderate*	< 0.001†
EED (m per min)	-0.37	(-0.74, 0.01)	*small*	0.054	-1.31	(-1.72, -0.90)	*large*	< 0.001†	-0.88	(-1.28, -0.48)	*moderate*	< 0.001†
Sprints (cases per min)	-0.60	(-0.98, -0.21)	*small*	0.001†	-1.18	(-1.59, -0.77)	*moderate*	< 0.001†	-0.59	(-0.98, -0.21)	*small*	< 0.001†
Jumps (cases per min)	-0.04	(-0.42, 0.33)	*trivial*	1.000	-0.75	(-1.14, -0.36)	*moderate*	0.142	-0.60	(-0.99, -0.21)	*moderate*	0.243
Exertions (cases per min)	-0.72	(-1.11, -0.33)	*moderate*	0.021†	-1.85	(-2.30, -1.41)	*large*	< 0.001†	-1.14	(-1.56, -0.73)	*moderate*	< 0.001†

**Internal measures**												
sRPE (AU)	0.46	(0.07, 0.84)	*small*	0.005†	-0.74	(-1.13, -0.35)	*moderate*	< 0.001†	-1.20	(-1.62, -0.79)	*laege*	< 0.001†
RPE (AU)	0.58	(0.20, 0.96)	*small*	0.003†	0.35	(-0.03, 0.73)	*small*	1.000	-0.15	(-0.53, 0.23)	*trivial*	0.032†

AAL = accumulated acceleration load; EED = estimated equivalent distance; sRPE = session rating of perceived exertion; RPE = rating of perceived exertion; AU = arbitrary units.

† Significant (*p* < .05) difference.

A repeated measures ANOVA revealed significant differences between groups for sRPE, *F*_(2, 141)_ = 15.74, *p* < .001 and RPE, *F*_(2, 141)_ = 5.23, *p* = .006. Post-hoc testing showed that after games against the Pro, significantly lower sRPE and RPE values were reported than after games against the Collegiate (d = 0.46, small, *p* = .005; d = 0.58, small, *p* = .003), whereas games against the Pro produced a significantly higher sRPE than the games against the Scrimmage (d = -0.74, moderate, *p* < .001).

## DISCUSSION

This study is the first to identify the differences in physical match demand due to the influence of the opponents in basketball. We found that, in general, as the competition level of the opponents increased, the relative external demands of the participants also increased. Contrarily, the internal response measured by sRPE for the study participants was lower after games against the Pro than after games against the Collegiate.

Game sports are unique events that involve dynamic interactions between players. As a result, the observed behaviour of an athlete or team is influenced by the situation or opponent [30]. In this study, no difference in EED was observed in participants during games against the Pro and Collegiate. Previous studies have reported that elite basketball athletes cover, on average, less distance than subelite and youth players [31]. In addition, high-level basketball players have explosive capacities such as sprinting and jumping [32], and this difference in neuromuscular capacity was considered to be reflected in the AAL. AAL is a cumulative measure of impact load in the triaxial direction, and this study revealed that participants had a higher impact load during matches against the Pro compared to during those against the Collegiate and Scrimmage. The differences in AAL measured in professional and collegiate matches may reflect differences other than running, i.e., differences in explosive high-intensity movements.

Petway et al. [31] reported that top-level basketball players spend more time performing high-intensity movements than do sub-elite players. The results of this study showed that the number of sprints and exertions of high-intensity movements increased with the competition level of the opponents. A previous study has shown that the external and internal game workloads vary depending on the contextual factors [20]. For example, losses may be more physically demanding than wins during basketball gameplay [20]. In this study, the participants lost all the matches against the Pro. Losing teams encounter an increased work rate due to a faster game pace when attempting to maximize the opportunities to score points and reduce the score-line [20]. Therefore, the higher AAL values measured during games against the Pro in this study indicate an increase in high-intensity movements performed, especially sprints and exertions.

On the other hand, in the games against the Pro, RPE values were significantly lower, and therefore, sRPE values were also lower, even though there was no difference in playing time. The low RPE value, despite the high level of competition of the opponents, may have been due to psychological factors. According to previous studies that measured sRPE during competition, perceptual measures might be influenced by psychological factors such as stress and anxiety associated with competition [33]. In turn, this might influence how the players perceive exertion, regardless of the physiological stress they are undergoing. Previous studies have shown that sRPE is higher in balanced games (with an end-margin of ≤ 8 points) than in unbalanced games (with an end-margin > 8 points) [20]. In this study, the Collegiate teams were from the same conference as the participants, and although it was a friendly match, there were many close games. As a result, the high psychological stress may have resulted in higher RPE. On the other hand, in the matches against the Pro, although the external load was recorded as high, there were fewer close games, and as a result, there was probably less psychological stress. In addition, in the matches against the Pro, the internal load was lower because the players felt positive towards the challenges according to their introspection reports. According to a previous study, subjective measures may also be more sensitive and consistent than objective measures [34]. Therefore, it is important to use an integrated approach to training loads, with a combination of internal and external training loads, which provides greater insight into training stress.

The results of games played against the Scrimmage showed that each of the variables of external load measured by the accelerometers was lower than that recorded for games against the Pro and Collegiate. This means that the external load was lower during training than during competitive matches, a finding that agrees with those of previous studies [35, 36]. To adequately prepare athletes for games, it is important to match the load (quantity and intensity) of the games through training at specific times during the preparation and competition phases. Furthermore, training should include preparation for worst-case scenarios in a match [37]. The external load intensities of basketball training drills substantially vary depending on the load indicator chosen, the training content, and task and individual constraints [38]. This study’s findings regarding the external demands that arise from playing against the Pro and Collegiate could help to guide scrimmage- and game-based drills. For example, the coaching staff can serve as a reference for the load criteria for game-based drills that assume games against higher-ranked opponents cannot be played frequently.

This study has several limitations. First, several elite leagues do not allow technology to be worn during competitions [31]. As a result, the games against the Pro and Collegiate in this study were unofficial. Our findings are relevant to friendly matches and cannot be generalized to official matches. Second, considering the application of the results of this study to training, there may be differences in playing position, which were not considered in this study. In future studies, the number of participants should be increased to clarify the position characteristics. Third, there is a lack of clarification and consensus across different tracking systems and manufacturers on how signals are filtered, calculations performed, or the suitable thresholds for basketball [7]. In this study, the same participants were fitted with the same accelerometers, allowing comparisons between performances against different opponents to be made. Future research is needed to synchronize acceleration data with videos of various event stamps, such as sprints, jumps, and exertion, to identify actual movements.

### Practical Application

Our findings provide important practical insights for basketball coaching staff, sports scientists, and players that can be used in various ways. First, AAL might be an optimal approach for quantifying basketball-specific high-intensity movements that cannot be measured by EED. It is inferred that the differences in AAL by competition level reflect differences in movements other than running, that is, explosive high-intensity movements. Second, an integrated approach to training load is important, which provides greater insight into training stress. In the present study, internal response (sRPE) was low despite the higher external workload in the games against the Pro. Thus, external and internal game loads should be monitored with the understanding that they may vary with each other depending on the contextual factors.

## CONCLUSIONS

This study examined the differences in physical game demands due to the influence of opponents in basketball. The results showed that as the competitive level of the opponents increased, the relative external load of the participants also increased. AAL is a useful indicator of the external load that reflects the competition level of opponents. In contrast, internal responses as measured by sRPE were lower after games against the Pro than after games with the Collegiate. In summary, it is important to use a combination of internal and external loads and monitor them with the understanding that they may vary with each other depending on the contextual factors. This allows for a better understanding of the stresses of training.
